# Use of DALYs in economic analyses on interventions for infectious diseases: a systematic review

**DOI:** 10.1017/S0950268814001940

**Published:** 2014-12-12

**Authors:** A. J. J. M. OOSTVOGELS, G. A. DE WIT, B. JAHN, A. CASSINI, E. COLZANI, C. DE WAURE, M. E. E. KRETZSCHMAR, U. SIEBERT, N. MÜHLBERGER, M.-J. J. MANGEN

**Affiliations:** 1 University Medical Centre Utrecht (UMCU), Utrecht, The Netherlands; 2 Academic Medical Centre (AMC), Amsterdam, The Netherlands; 3 National Institute for Public Health and the Environment (RIVM), Bilthoven, The Netherlands; 4 Institute of Public Health, Medical Decision Making and Health Technology Assessment, Department of Public Health and Health Technology Assessment, UMIT – University for Health Sciences, Medical Informatics and Technology, Hall i.T., Austria; 5 Division of Health Technology Assessment and Bioinformatics, ONCOTYROL – Center for Personalized Cancer Medicine, Innsbruck, Austria; 6 European Centre for Disease Prevention and Control (ECDC), Stockholm, Sweden; 7 Institute of Public Health, Catholic University of the Sacred Heart, Rome, Italy; 8 Center for Health Decision Science, Department of Health Policy and Management, Harvard School of Public Health, Boston, MA, USA; 9 MGH-Institute for Technology Assessment and Department of Health Policy and Management, Harvard University, Boston, MA, USA

**Keywords:** Cost-effectiveness analysis, costs, disability-adjusted life years (DALYs), economic evaluation, infectious diseases, systematic review

## Abstract

A systematic literature review was performed on full economic evaluations of infectious disease interventions using disability-adjusted life years (DALY) as outcome measure. The search was limited to the period between 1994 and September 2011 and conducted in Medline, SciSearch and EMBASE databases. We included 154 studies, mostly targeting HIV/AIDS and malaria with most conducted for African countries (40%) and <10% in high-income countries. Third-payer perspective was applied in 29% of the studies, 25% used the societal perspective and 12% used both. Only 16% of the studies took indirect effects (i.e. herd immunity) of interventions into account. Intervention, direct healthcare and indirect non-healthcare costs were taken into account in respectively 100%, 81% and 36% of the studies. The majority of the studies followed the Global Burden of Disease method for DALY estimations, but most studies deviated from WHO cost-effectiveness guidelines. Better adherence to freely accessible guidelines will improve generalizability between full economic evaluations.

## INTRODUCTION

The disability-adjusted life year (DALY) methodology, which was jointly developed for the Global Burden of Disease (GBD) study by the World Bank, the World Health Organization (WHO) and the Harvard School of Public Health in the late 1980s [1–5], measures both mortality and morbidity and combines them in one single figure, allowing the comparison of health hazards and providing an evidence-based tool for healthcare policy prioritization and for monitoring intervention effects. Since its development, the DALY measure has been used widely in both national and global disease burden (e.g. [6–9]) and cost-effectiveness studies (e.g. [10–12]). The WHO recommends the use of DALYs in cost-effectiveness studies for the purpose of comparability [13, 14]. DALY losses and costs are estimated for each intervention under study and then compared using the incremental cost-effectiveness ratio (ICER) to determine which intervention will offer the best value for money invested [13]. A specific property of infectious diseases that distinguishes them from chronic diseases is that infected persons who are treated may not only be cured (i.e. curative intervention) or protected against an infection (i.e. preventive intervention such as vaccination), but also that a successful intervention might reduce or prevent transmission of the pathogen to other susceptible persons [15]. Consequently, the force of infection acting on those individuals who remain susceptible changes, a phenomenon that is known as herd immunity (or indirect intervention effect). If herd immunity has a large impact on transmission, dynamic transmission models are recommended for health economic analyses [14, 16, 17]. Another property of infectious diseases is that infections do not only lead to acute illness but might also result in chronic and long-term sequelae, requiring the use of an incidence- and pathogen-based DALY approach [18, 19].

The WHO has issued guidelines to enhance the comparability of cost-effectiveness measures of different interventions (hereafter referred to as WHO-CEA guidelines) [13]. In addition, several countries and scientific organizations have published guidelines for economic evaluation. Reviews have shown that these guidelines are not always followed properly (e.g. [20–24]). In order to examine how the specific WHO guidelines (i.e. WHO-CEA guidelines [13] and GBD methodology [1, 3, 25, 26]) are followed in economic evaluations of preventive and therapeutic interventions for infectious diseases using DALYs as an effectiveness parameter, we conducted a systematic literature review.

## METHODS

### Search strategy

Medline, EMBASE and SciSearch were searched starting from 1994 (the year after the introduction of the DALY concept in the World Development Report [27]) up to 1 September 2011. The search syntax (see online Appendix I) combined infectious disease-related terms (both general terms and particular pathogens/diseases) and DALY-related terms. As we expected to find few hits using these terms, no further restrictions (i.e. economic-related terms) were imposed at this stage.

### Eligibility criteria

Only economic evaluations of infectious disease interventions considering both costs and effects (i.e. DALYs) of two or more alternatives and comparing the alternatives based on incremental analysis [13] were eligible. Furthermore, we limited our search to papers written in English, Spanish, German, French, Italian or Dutch.

### Screening

Abstracts, title and key words were read by two reviewers (A.O. and M.-J.J.M.) to identify papers that appeared to fulfil the eligibility criteria. Review papers were also identified and checked for potential references. In the event of disagreement between reviewers, abstracts were double-checked and discussed until consensus was reached. Full-text articles were retrieved and read if the abstracts met the eligibility criteria or if these were identified via reference lists of papers already identified.

### Data extraction

Using a pre-specified data extraction form, which was drawn up prior to data extraction and agreed upon by all authors, 55 items (see Table 1) were extracted in a standardized manner for each identified economic evaluation by two reviewers independently. Extracted data included study identifiers, disease/pathogen, intervention and comparator, costs, health outcomes, type of model used (if any), economic evaluation characteristics, as well as key parameters used for sensitivity analyses (Table 1). Items were selected to check if studies followed the GBD study when estimating DALYs (i.e. using GBD disability weights, applying age-weighting, a 3% discount rate; standard life expectancy) and whether economic evaluations were conducted accordingly to WHO-CEA guidelines [13]. As our review was finalized in September 2011, we have not considered adherence to the recent update of GBD methodology [28]. In the event of disagreement between reviewers, items were double-checked and discussed until consensus was reached.

**Table 1.** Data extraction^*a*^

Type of characteristics	List of items extracted
Study identifiers	• Name of first author
	• Year of publication
	• Name of journal
	• Journal type
	• Does one of the authors have an affiliation with the industry?
	• Article type (e.g. research article, review)
	• Country name
	• Geographical area
	• Language of paper
Pathogen/disease and burden of disease	• Pathogen/disease
	• Target population (e.g. single cohort, multiple cohorts), and age group
	• Listing health outcomes and specify if, and which sequelae were taken into account
	• Use of incidence or prevalence data
	• Use of pathogen- or outcome-based approach
	• Other than DALY metrics used? If yes, which?
	• Which life expectancies were used?
	• Competing background mortality taken into account?
	• Which disability weights were used?
	• Was age-weighting applied?
	• Was gender-weighting applied?
	• Base-case discount rate for effects
Intervention, effect and model	• Intervention type (e.g. vaccination; screening)
	• Type of technology used for intervention (e.g. mechanical, medical, education)
	• Transmission route considered
	• Direct and/or (if then applicable) indirect effects considered
	• Sector where intervention occurred
	• Which alternative(s) were studied?
	• Use of real data (e.g. randomized clinical trial)
	• Use of transmission model, and details
	• Use of Markov model, and details
	• Use of economic model
	• Use of Monte Carlo simulation technique (single)-cohort/population-based model
Economic evaluation	• Perspective reported and defined^a^
	• Costs categories considered^b^
	• Category of productivity losses considered (e.g. absenteeism for patients and/or caregivers)
	• Use of human capital or the friction cost approach for estimating productivity losses
	• Time horizon considered (in years)
	• Year for which the costs were estimated
	• Currency used
	• Cost per DALY
	• Other cost-effectiveness given? If yes, which one?
	• Intervention cost-effective according to the authors
	• If given, list the threshold used for the cost-effectiveness decision. Was this the same threshold as suggested by the WHO and as estimated^c^?
	• Base-case discount rate for costs
Sensitivity analysis (SA) performed	• SA performed
	• Type of SA performed (e.g. one-way SA, multi-way SA, probabilistic SA)
	• SA for incidences
	• SA for simulated effects of intervention
	• SA for DALYs (e.g. disability weights, life expectancy, etc.)
	• SA for intervention itself
	• SA for costs modeled
	• SA for discount rates used (effects/costs)

a We noted the perspective as reported by the authors (i.e. stated perspective), and based on cost categories considered, and following the criteria listed in Table 2, we defined the perspective ourselves (see Table 2 for definition used, and Appendix II for details).

b We distinguished the following cost categories: (i) intervention costs (IC); (ii) averted direct healthcare costs (DHC) such as averted medical service costs due to averted disease; (iii) averted indirect non-healthcare costs (INHC) which were mainly averted productivity losses due to e.g. reduced absence from work; (iv) averted direct non-healthcare costs (DNHC) such as averted costs by patients for averted travelling, etc.

c We looked up the local *per capita* Gross Domestic Product for the study year and the currency applied, and estimated the threshold according to the recommendation made by the WHO for the corresponding year using the following website: http://data.worldbank.org/indicator (accessed 7 October 2013).

### Data analysis

Using the data extraction form, frequencies for the different study characteristics were calculated. Based on the cost categories considered and following the criteria listed in Table 2, for each study, we defined the perspective and compared them with the stated perspective (for details see online Appendix II). Additionally, for each study, we estimated the year and country-specific cost-effectiveness thresholds as suggested by the WHO-CEA guidelines [i.e. one times the *per capita* Gross Domestic Product (GDP) is considered highly cost-effective, three times GDP is considered cost-effective].

**Table 2.** Defined perspective by authors based on cost categories considered

Perspective	Cost categories considered^a^
Societal perspective^b^	IC and DHC and DNHC and INHC
Third-payer perspective^b^^,^^c^	IC and DHC
Programme perspective^b^	IC
Provider perspective^b^^,^^d^	IC and INHC
Limited societal perspective^e^	IC and DHC and INHC
Limited societal perspective^e^	IC and DHC and DNHC

a IC, Intervention costs; DHC, direct healthcare costs; DNHC, direct non-healthcare costs (also referred to as out-of-pocket costs or patient costs); INHC, indirect non-healthcare costs (mainly only productivity losses, but also costs such as special education).

b Based on economic textbooks such as Drummond *et al.* [44] and Gold *et al*. [45].

c Third-payer perspective or healthcare-payer perspective.

d Two of the analysed studies used as perspective, the working company.

e If three of the four cost categories from a societal perspective were considered we marked them as limited societal perspective.

## RESULTS

### Literature review

**Fig. 1.**
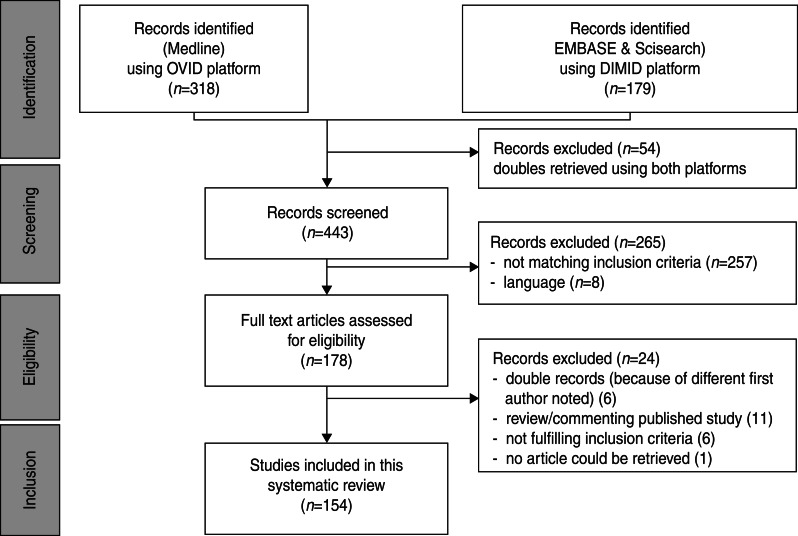
Study flowchart.

We identified 497 hits (a list of all excluded articles is available on request from the corresponding author. A list of all included articles is given in Appendix II). After deleting double entries, 443 abstracts were screened. Abstracts of papers not written in English, Spanish, German, French, Italian or Dutch (*n* = 8) and abstracts not meeting the inclusion criteria (*n* = 257) were excluded. Finally, 178 abstracts met the inclusion criteria. Full-text articles of 177 hits were available. The paper by Kinghorn [29] was not accessible. Six articles were excluded as double entries; six other papers did not meet the eligibility criteria. Eleven papers which either reviewed or commented on a single published study were excluded. Reviews and selected papers’ reference lists were checked for additional relevant references; however, no other papers could be identified. Eventually, 154 articles were included in the systematic review (see Fig. 1).

#### Study identifiers

**Fig. 2.**
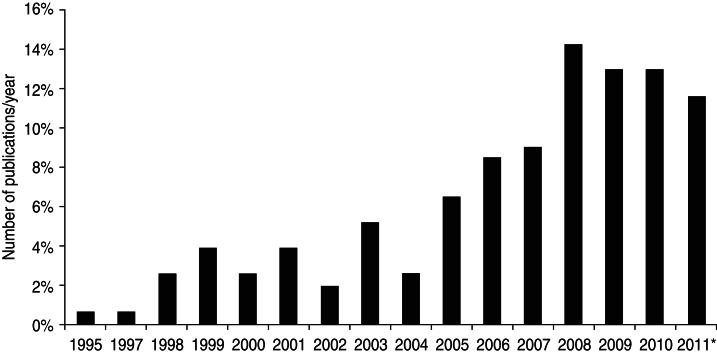
Annual number of published economic evaluations using DALYs as health outcome. * Number of papers in 2011 until 1 September.

The first economic evaluations on infectious diseases using DALYs were published in 1995 [30], but most studies were published in more recent years (2008–2011) (Fig. 2). Most studies were conducted for countries in Africa (40%), Asia (21%) or Latin American and the Caribbean countries (15%) and 14% of the articles were global, considering more than one continent. Few studies were conducted in North America (1·4%), Europe (5·3%) and Oceania (3·3%) (see Table 3). Only 3% of the included studies were in Spanish, all others were in English (97%).

**Table 3.** Geographical area and disease (disease category) studied

Disease/disease category	Geographical area					
	Africa	Asia	Latin American & Caribbean countries	High-income countries^a^	Global	Total
Parasitic diseases^b^	24	2	4	2	1	33
Vaccine-preventable diseases^c^	10	15	12	9	10	56
HIV/AIDS and other STDs^d^	22	7	3	1	4	37
Tuberculosis	1	5	4	1	2	13
Gastrointestinal diseases^e^	0	0	0	2	1	3
Zoonoses^f^	1	1	0	0	0	2
Multiple^g^	2	0	0	0	2	4
Other^h^	2	2	1	0	1	6
Total	62	32	24	15	21	154

a Europe, North America and Oceania.

b Malaria (22), echinococcosis (2), intestinal parasites (1), leishmaniasis (3), trypanosomiasis (5).

c Measles (4), tetanus (1), polio (4), *Haemophilus influenzae* type B (6), influenza (1), hepatitis B (3), typhoid fever (2), pneumonia (9), meningitis (2), Japanese encephalitis (3), cholera (2), pertussis (1), Rotavirus (18).

d Other sexually transmittable diseases (STDs) are: syphilis (5), human papillomavirus (2).

e *Campylobacter* (2), diarrhoea (1).

f Brucellosis (1), rabies (1).

g HBV, HCV and HIV infections (1), rotavirus and HPV vaccination (1), enhanced outreach system (i.e. vitamin A, vaccination, etc.) (1), water sanitation and malaria treatment and education (1).

h *Aspergillus flavus* (1), cryptococcal (1), dengue (3), trachoma (1).

#### Burden of disease

Overall, studies dealt with 29 different infectious diseases with four papers combining multiple diseases (Table 3). Interventions for HIV/AIDS were studied most frequently (20%), followed by interventions for malaria (14%) and rotavirus (12%). Of the studies conducted for Africa, one third concerned HIV/AIDS (67% of all HIV/AIDS studies) and one third concerned malaria (91% of all malaria studies).

Country-specific life expectancy was used in 63% of the studies. Standard life expectancy as used in the GBD study [3] was applied in 12% of the papers, 2% used both, while 4% based life expectancy on the literature and 2% explicitly modelled it. No information on life expectancy was provided in 17% of the papers. Most studies used GBD disability weights (72%), 5% used country-specific disability weights (i.e. Dutch and Australian disability weights), for 8% it was unknown which disability weights were used, 6% used other disability weights, and 9% considered only premature mortality as a disease burden as morbidity was considered marginal.† Age-weighting was reported to have been applied in 44% of the studies; 40% used no age-weighting and in 16% of the studies it was unknown if age-weighting was applied or not.

Acute illness and related sequelae were explicitly modelled in 38% of the studies. In 57% of the studies only one health outcome (not necessarily acute illness) was considered and in 5% of the studies it was unclear which disease stage was used. Note that not all infectious diseases result in the development of sequelae (e.g. rotavirus).

### Intervention, effect and model

#### Evaluated interventions

In 94% of the studies, either care-as-usual or do-nothing (i.e. situation where no intervention is applied, also referred by the WHO as ‘null set’) was used as the comparator. In the remainder of the studies, the intervention was compared with another treatment (3%), or both with do-nothing and another treatment (2%), or with a change in the vaccination strategy (1%). Most of the interventions analysed concerned vaccination (42%), followed by prevention measures other than vaccination (20%) and by treatment (20%) (Table 4). Most of the interventions evaluated were performed in healthcare settings (87%), followed by household settings (8%), agricultural/environmental settings (4%) or a combination of healthcare and household settings (1%).

**Table 4.** Disease studied and intervention under study in the 154 papers

Disease/disease category^a^	Interventions						Total
	Diagnosis	Prevention^b^	Screening	Treatment	Vaccination	Combination	
Parasitic diseases	1	10	1	13	2	6^c^	33
Vaccine-preventable diseases	0	0	0	0	55	1^d^	56
HIV/AIDS and other STDs	0	11	7	10	3	6^e^	37
Tuberculosis	3	0	2	6	1	1^f^	13
Gastrointestinal illness	0	3	0	0	0	0	3
Zoonoses	0	1	0	0	1	0	2
Multiple	0	3	0	0	1	0	4
Other	0	3	1	1	1	0	6
Total	4	31	11	30	64	14	154

a For more details on disease/disease category see Table 3 notes.

b Prevention other than vaccination.

c All six studies combined prevention and treatment as intervention.

d The interventions in this study consisted of vaccination and treatment.

e The interventions in these studies consisted of prevention (medical)/treatment/education (2), screening/prevention/treatment/screening (2), prevention and treatment (1), vaccination and treatment (1)

f The interventions in this study consisted of prevention and treatment.

#### Model characteristics

To estimate the disease burden, 92% of the papers used incidence data and 8% used prevalence data.

Primary data (i.e. economic evaluations using data from, e.g. randomized clinical trials, cohort studies) was used in 27% of the studies. The remaining studies used secondary data [i.e. economic evaluation gathering data from (systematic) reviews from literature], or a combination of both, to conduct their cost-effectiveness analysis.

In 27% of the studies, either a dynamic transmission/risk assessment model (16%) or a Markov model (11%) was used to support the analysis.

Most studies considered only direct intervention effects (84%), 12% modelled both direct and indirect intervention effects and 4% studied indirect intervention effects (i.e. herd immunity), but only as a sensitivity analysis.

The Monte-Carlo simulation technique was used in 59 papers (38%), of which four papers only were for their sensitivity analysis.

### Economic evaluation and methods

#### Costs and perspective

Intervention costs (IC) were taken into account in all studies, while direct healthcare costs (DHC), direct non-healthcare costs (DNHC) and indirect non-healthcare costs (INHC) were considered in 81%, 32% and 36% of the studies, respectively.

INHC was considered in 36% of the papers (i.e. 55 papers). Butler *et al*. [31] evaluated additional costs due to special education needs, all others (i.e. 54 studies) evaluated productivity losses due to work absence of either the patient, their caregiver or both. Most studies (74%) that considered work absence of caregivers were studies estimating the effect of child vaccination.

According to the authors, 38% of the studies used the third-payer perspective (i.e. IC and DHC), of which two also took the programme perspective (i.e. IC), 24% used a societal perspective (i.e. IC, DHC, DNHC, INHC) and 15% used both, third-payer and societal, perspective (except for one paper that used the programme and societal perspective). In 17% of the studies, no perspective was specified and 6% used other perspectives (e.g. company perspective).

With regard to the method used to value productivity losses: three studies (5·5%) stated that they used the human capital approach. The friction costs approach was stated to have been used in three Dutch studies (5·5%), according to Dutch guidelines [32]. The other studies did not specify which method they used.

Authors claiming to have taken a third-payer perspective (i.e. 45 papers) should at least have considered both IC and DHC, but 13% of the analysed studies only considered IC. Moreover, only 50% of the studies claimed to have used a societal perspective, in total 38 papers had included all costs that should be considered in this perspective (i.e. IC, DHC, DNHC, INHC) (Appendix II). Of the 123 papers that stated their perspective explicitly, 87 used the appropriate costs according to the perspective chosen (70%).

Less than half of the studies (44%) used only one single ICER, namely cost/DALY averted. In the other 56% of studies, additional ratios were shown. This was mostly cost/life year gained. But also physical units such as, e.g. averted infections, lives saved or averted hospitalizations were used as effect measures.

The cost-effectiveness threshold used to underpin their assessment of cost-effectiveness was listed in 74 studies (48%). The majority of them (96%) adopted the WHO-CEA guidelines and 4% used (unofficial) country-specific thresholds. Despite the fact that not all studies stated the cost-effectiveness threshold used, 87% of all studies came to a conclusion favouring the cost-effectiveness of the interventions, namely cost-effective (49%), highly cost-effective (33%) and cost-saving (5%). Only 7% reported that the interventions were not cost-effective and 2% did not state whether the interventions were cost-effective. The remaining 4% studied multiple countries/interventions with different conclusions for countries/interventions.

### Time horizon and discounting

Most of the studies (98%, *n* = 151) had a time horizon longer than 1 year and should have applied discounting according to the guidelines [13]. Of these 151 studies, 83% discounted the costs, 7% did not mention discounting and 10% did not discount the costs (sometimes because all costs were incurred in a single year). If costs were discounted (*n* = 125), the discount rate used was most often 3% (86%). The other 17 studies used 2% (*n* = 1), 3·5% (1), 4% (4) 5% (8), 10% (2) and 15·8% (1). A sensitivity analysis with regard to the discount rate for costs was performed in 47% of the studies (*n* = 59).

Health effects (i.e. DALYs) were discounted in 92% of the studies. Of the remaining 12 studies, 11 papers did not provide any information on discounting and one paper stated explicitly that the effects had not been discounted. If the effects were discounted (*n* = 142), the discount rate used most often was 3% (92%). The other 11 papers used a discount rate of 1·5% (*n* = 2), 3·5% (1), 4% (2) and 5% (6). A sensitivity analysis on the discount rate of the effects was performed in 47% of the papers (*n* = 67).

Both costs and effects were discounted in 124 studies (81%). Equal rates of discounting for both costs and effects were applied in 94% of these papers, 5% of these papers used lower discount rates for effects than for costs and 1% vice versa.

### Sensitivity and uncertainty analyses

A sensitivity analysis was performed and described in 97% of all studies. One-way sensitivity analysis was applied in 46% of those studies. Other deterministic sensitivity analyses (two-way, multi-way, threshold or combination of these) were performed in 30% of the studies. A combination of deterministic and probabilistic sensitivity analyses were used in 3% of the studies, while 13% of the studies reported a probabilistic approach only. For the remaining 8%, it was unclear what kind of sensitivity analysis was performed. Sensitivity analyses were mainly performed on parameters regarding the effects of the intervention (89%), followed by the incidence of the disease (82%), the costs of the intervention (80%) or the form of the intervention (e.g. two or three dose vaccination strategies) (77%). Only 29% of the studies performed a sensitivity analysis on the calculation of DALYs (e.g. disability weights used, with and without age-weighting).

### Adherence to guidelines

The adherence to guidelines on cost-effectiveness analysis was low for some criteria, but higher for others (Table 5). Authors adhered to the guidelines most frequently in the use of sensitivity analyses (97%), use of GBD disability weights (72%) and use of a 3% discount rate for both costs and effects, where appropriate (72%). The use of age-weighting when calculating DALYs was only applied in 44% of the studies. WHO life expectancy was used only in 14% of the studies.

**Table 5.** Adherence to WHO-CEA guidelines

Criteria	Adherence, *n* (%)					
	Total (154)	Africa (62)	Asia (32)	Latin American & Caribbean countries (24)	High-income countries (15)^a^	Global (21)
Sensitivity analysis applied	149 (97%)	59 (95%)	31 (97%)	24 (100%)	15 (100%)	20 (95%)
GBD disability weights used	111 (72%)	42 (68%)	23 (72%)	18 (75%)	7 (47%)	21 (100%)
3% discount rate, if then appropriated	107 (69%)	38 (61%)	28 (88%)	21 (88%)	3 (20%)	17 (81%)
Age-weighting applied	67 (44%)	29 (47%)	14 (44%)	12 (50%)	2 (13%)	10 (48%)
WHO Standard life expectancy	21 (14%)	7 (11%)	6 (19%)	3 (13%)	1 (7%)	4 (19%)
All criteria	6 (4%)	2 (3%)	2 (6%)	0 (0%)	0 (0%)	2 (10%)
Use of a cost-effectiveness threshold	74 (48%)	28 (45%)	18 (56%)	12 (50%)	5 (33%)	11 (52%)
Perspective stated	118 (77%)	45 (73%)	24 (75%)	20 (83%)	13 (87%)	16 (76%)
Stated and defined perspectives were the same	73 (47%)	23 (37%)	15 (47%)	15 (63%)	10 (67%)	10 (48%)

a Europe, North America and Oceania.

## DISCUSSION

We identified 154 economic evaluations on infectious disease interventions that used DALYs as a health outcome measure, which were mostly conducted in the last decade. The quality of reporting was insufficient in many studies, the methodological choices were not all stated clearly; sometimes the choices that had been made had to be derived from references to other articles about the methodology used.

With more than 10% of the evaluated studies being performed outside the healthcare setting, a wider literature search than medical databases is a must in case of infectious diseases. Although we found studies from all continents, this review showed that most economic evaluations using DALYs for infectious disease interventions were conducted for low-income countries. This could be related to the fact that guidelines for economic evaluations in high-income countries in general require the use of the more data-intensive quality-adjusted life years (QALYs) [14] as the standard health outcome. Whereas the WHO-CEA guidelines [13] recommend the use of DALYs. Another reason might be the fact that there are no, or only poor, utility weights available for infants and children [14], as most of the questionnaires used to derive QALYs are not suitable for children, and even less so for infants. More than 40% of the economic evaluations that were reviewed evaluated vaccination interventions targeting young children.

The studies included in the review seldom considered the full effect of an intervention. For instance, in most studies on acute illness the focus was related to a particular pathogen only, and not on the long-term sequelae of that pathogen. However, not considering sequelae can result in a significant underestimation of the true disease burden and therefore of the clinical and economic consequences of an intervention [18, 19, 33, 34]. Moreover, indirect intervention effects (herd immunity effects) were seldom covered. However, considering or not considering indirect effects could lead to different conclusions (see e.g., Jeuland *et al*. [35] who presented the cost-effectiveness ratios with and without herd immunity). According to ISPOR guidelines on good modelling practices [17] dynamic models are important in two circumstances, namely (1) when an intervention impacts a pathogen's ecology, and (2) when the intervention impacts disease transmission. On the other hand, if these restricted studies proved to be cost-effective without omitting significant negative side-effects, more complex models are seldom expected to change these conclusions. On the contrary, they would most likely report even more favourable cost-effectiveness ratios. It is therefore always a trade-off between building a more complex model in order to obtain more detailed results *vs.* a simplified model that is faster to build, less data intensive and potentially easier to understand and communicate.

In the current review, most interventions proved to be cost-effective, and – according to modelling guidelines [17] – there is no need for more complex models if it is certain that negative dynamic effects can be ruled out. Only 11 studies reported that the analysed intervention was not cost-effective. Of the studies reporting that the interventions were not cost-effective, in a third of these studies the intervention had no impact on disease transmission and therefore dynamic modelling would not have been appropriate (e.g. irradiation of chicken meat at the end of the slaughter line to reduce the *Campylobacter* load). A quarter of these studies had considered the impact on herd immunity either by using dynamic modelling or conducting sensitivity analysis. But in the remaining studies, dynamic modelling would have been necessary to obtain the full effect and hence, the full cost-effectiveness, of the interventions analysed.

Only six of the 54 studies estimating productivity losses had explicitly stated the applied methodology, whereas 50% used the human-capital method and 50% the friction cost-method. Most of the other studies considered productivity losses related to work absence during a temporary illness of either the patient himself and/or his caregiver and most likely used the human-capital method to value productivity losses, without stating this explicitly. A few studies had also additionally considered costs for ‘forgone non-market activities including school, housework and childcare’ (e.g. Cook *et al*. [36] or Jeuland *et al*. [35]), or ‘lost earning avoided due to morbidity and premature mortality’ [37], or ‘lifetime pension for patients developing life-long disabilities’ [38]. These studies definitively used the human-capital approach. In the case of short-term absenteeism (up to half a year), both the human-capital and the friction cost-method will lead to identical results. However for long-term absenteeism, disability and premature mortality, the human-capital method will always estimate far higher cost differences than the friction cost-method [39], and this will consequently result in more favourable cost-effectiveness ratios than when using the friction cost-method. For this reason, we conclude that clear communication on the method used is necessary to fully appreciate study results.

WHO-CEA guidelines recommend ICER thresholds based on the *per capita* GDP. The majority of studies followed these guidelines. However, economic evaluations using a societal perspective – and therefore considering also productivity losses – tend to have a more favourable ICER (e.g. [40–42]) than for example when using a third-payer perspective. For this reason, we conclude that clear communication on the perspective used and more directive guidelines regarding cost-effectiveness and the perspective to be used are necessary to fully appreciate study results, to compare them with other studies and to assess the level of cost-effectiveness of interventions.

This review makes it clear that – similar to other reviews on economic evaluations (e.g. [25, 26]) – many authors who have conducted a full economic evaluation on infectious diseases using DALYs as a health outcome, adhere to general economic principles and clearly describe the methodology in their papers. However, many others do not provide sufficient information for an assessment of the applied methodology, and some authors even claim more than what was carried out. Therefore, the systematic use of more standardized guidelines such as the newly published Consolidated Health Economic Evaluation Reporting Standards (CHEERS) guidelines [43] would increase the comparability of the results across studies in different countries worldwide.

## Footnotes

† These studies have only considered mortality as the single health outcome, therefore it is disputable whether these studies should be considered at all in the current review. However, we took them into consideration as the authors claimed that morbidity was marginal and therefore negligible.

## Supplementary material

For supplementary material accompanying this paper visit http://dx.doi.org/10.1017/S0950268814001940. click here to view supplementary material
