# Supplementary material for: Use of DALYs in economic analyses on interventions for infectious diseases: a systematic review
Source: Epidemiol Infect. 2014 Dec 12;143(9):1791–802. doi: 10.1017/S0950268814001940 (PMC4456769; doi:10.1017/S0950268814001940)
Supplement: Supplementary file 1 [file S0950268814001940sup.zip › S0950268814001940sup001.doc]

**Supplementary Material**

**Appendix I – Applied search strategy**

### *Searching for literature using two different platforms.*

Note: We used general terms for infectious diseases, and additionally searched by name for pathogens/diseases listed on the surveillance list of ECDC, but without excluding infectious diseases not under ECDC surveillance. The search was not case-sensitive.

### *A.1) Search strategy in OVID Medline:*

| **Ovid MEDLINE(R), Ovid MEDLINE(R) In-Process** | | | |
| --- | --- | --- | --- |
| **#** | **Searches** | **Results** | **Search Type** |
| 1 | exp bacterial infections/ or exp virus diseases/ or exp parasitic diseases/ or exp mycoses/ or exp central nervous system infections/ or exp zoonoses/ or exp communicable diseases/ or exp infection/ | 1777384 | Advanced |
| 2 | (haemophilus influenzae type b or hemophilus influenzae type b or hib or h influenzae type b or influenza or influenzavirus* or measles or rubeola or morbilli or mumps or infectious parotitis or rubella or pertussis or whooping cough or poliomyelitis or polio or poliovirus or tetanus or diphtheria or meningococc* or pneumococc* or meningitidis or streptoccocus pneumoniae or pneumoniae or ipd or pnsp or human papilloma* or hpv or vaccine preventable diseases).ti. | 152282 | Advanced |
| 3 | (smallpox or variola or anthrax or bacillus anthracis or botulism or clostridium botulinum or brucellosis or brucella or malta fever or mediterranean fever or echinococcosis or echinococciasis or echinococcus or q fever or coxiella burnetii or c burnetii or rabies or tularaemia or rabbit fever or francisella tularensis or f tularensis or west nile fever or wnf or west nile virus or haemorrhagic fever* or hemorrhagic fever* or vhf or cchf or hantavirus or rickettsia* or lyme disease or borrelia or burgdorferi or malaria or marsh fever or plasmodium).ti. | 104816 | Advanced |
| 4 | (chlamydia or chlamydia trachomati or c trachomatis or gonococcal infection* or gonorrh* or syphilis or treponema pallidum or t pallidum or hiv or aids or (acquired adj3 deficiency syndrome) or human immunodeficiency virus or hepatitis a or hav or hepatitis b or hbv or hepatitis c or hcv).ti. | 288902 | Advanced |
| 5 | (campylobacter or cryptosporidiosis or cryptosporidium or giardiases or giardia lamblia or leptospirosis or weil disease or weils disease or leptospira or listeriosis or listeria or salmonella or salmonellosis or salmonella enteritidis or s enteritidis or salmonella typhimurium or s typhimurium or shigella or shigellosis or bacillary dysentery or toxoplasmosis or toxoplasma or t gondii or trichinosis or trichinella spiralis or t spiralis or yersinia or yersiniosis or y enterocolitica or escherichia coli or e coli or shiga toxin* or vero toxin* or vtec or stec or enterococc*).ti. | 189764 | Advanced |
| 6 | (creutzfeldt-jakob or creutzfeldt jakob or vcjd or variant cjd or transmissible spongiform or legionellosis or legionnaires' disease or legionella pneumophila or l pneumophila or Severe acute respiratory syndrome or sars or klebsiella pleumoniae or k pneumoiae or pseudomonas aeruginosa or p aeruginosa or aureus or mrsa or staphylococc* or tuberculosis or tb or mdr tb or mdr-tb or multidrug-resistant tb).ti. | 170566 | Advanced |
| 7 | ((waterborne adj5 disease*) or (soilborne adj5 disease*) or (airborne adj5 disease*) or (foodborne adj5 disease*) or food infection or food poisoning or sexually transmitted diseases or std or sexually transmitted agents or emerging infectious disease* or infectious disease* or communicable disease* or respiratory virus infections or respiratory viral infections or respiratory infections).ti. | 22543 | Advanced |
| 8 | (direct contact transmission or (person-to-person adj5 transmission) or (person-to-person adj5 disease*) or (animal-transmitted adj5 disease*) or (arthropod-transmitted adj5 disease*) or zoonoses or zoonosis).ti. | 1289 | Advanced |
| 9 | 1 or 2 or 3 or 4 or 5 or 6 or 7 or 8 | 2087657 | Advanced |
| 10 | (disability adjusted or daly or dalys or "daly’s").tw. | 1119 | Advanced |
| 11 | 9 and 10 | 329 | Advanced |
| 12 | remove duplicates from 11 | **318** | Advanced |

### *A.2) Combined search strategy in Medline, Embase & SciSearch using the platform DIMDI*

3 databases	with 71118700 documents selected:

1. ME66	MEDLINE	NLM
2. EM74	EMBASE	2011 Elsevier B.V.
3. IS74	SciSearch	Thomson Reuters

C=	1 71118700 ME66; EM74; IS74

S=	2	3402552 CT D (BACTERIAL INFECTIONS; BACTERIAL INFECTION; VIRUS DISEASES; VIRUS INFECTION; PARASITIC DISEASES; PARASITOSIS; MYCOSES; MYCOSIS; CENTRAL NERVOUS SYSTEM INFECTIONS; ZOONOSES; COMMUNICABLE DISEASES; COMMUNICABLE DISEASE; INFECTION)

3	348345 FT=(HAEMOPHILUS INFLUENZAE TYPE B; HEMOPHILUS INFLUENZAE TYPE B;HIB; H INFLUENZAE TYPE B; INFLUENZA; INFLUENZAVIRUS?; MEASLES; RUBEOLA; MORBILLI; MUMPS; INFECTIOUS PAROTITIS; RUBELLA; PERTUSSIS; WHOOPING COUGH; POLIOMYELITIS; POLIO; POLIOVIRUS; TETANUS) / (TI;CT;UT)

4	263153 FT=(DIPHTHERIA; MENINGOCOCC?; PNEUMOCOCC?; MENINGITIDIS; STREPTOCCOCUS PNEUMONIAE; PNEUMONIAE; IPD; PNSP; HUMAN PAPILLOMA?; HPV; VACCINE PREVENTABLE DISEASES) / (TI;CT;UT)

5	178225 FT=(CRIMEAN-CONGO HEMORRHAGIC FEVER VIRUS; LASSA FEVER; HANTAVIRUS INFECTIONS; HANTAVIRUS; LYME DISEASE; BORRELIA BURGDORFERI; MALARIA) / (TI;CT;UT)

6	143498 FT=(SMALLPOX; VARIOLA; ANTHRAX; BACILLUS ANTHRACIS; BOTULISM; CLOSTRIDIUM BOTULINUM; BRUCELLOSIS; BRUCELLA; MALTA FEVER; MEDITERRANEAN FEVER; ECHINOCOCCOSIS; ECHINOCOCCIASIS; ECHINOCOCCUS; Q FEVER; COXIELLA BURNETII; C BURNETII; RABIES; TULARAEMIA) / (TI;CT;UT)

7	259958 FT=(FRANCISELLA TULARENSIS; F TULARENSIS; WEST NILE FEVER; WNF; WEST NILE VIRUS; HAEMORRHAGIC FEVER?; HEMORRHAGIC FEVER?; VHF; CCHF; HANTAVIRUS; RICKETTSIA?; LYME DISEASE; BORRELIA; BURGDORFERI; MALARIA; MARSH FEVER; PLASMODIUM; S TYPHIMURIUM) / (TI;CT;UT)

8	1213074 FT=(CHLAMYDIA; CHLAMYDIA TRACHOMATIS; C TRACHOMATIS; GONOCOCCAL INFECTION?; GONORRH?; SYPHILIS; TREPONEMA PALLIDUM; T PALLIDUM; HIV; AIDS; ACQUIRED DEFICIENCY SYNDROME; HUMAN IMMUNODEFICIENCY VIRUS; HEPATITIS A; HAV; HEPATITIS B; HEPATITIS C; HCV) / (TI;CT;UT)

9	279956 FT=(CAMPYLOBACTER; CRYPTOSPORIDIOSIS; CRYPTOSPORIDIUM; GIARDIASES; GIARDIA LAMBLIA; LEPTOSPIROSIS; WEIL DISEASE; WEILS DISEASE; LEPTOSPIRA; LISTERIOSIS; LISTERIA; SALMONELLA; SALMONELLOSIS; SALMONELLA ENTERITIDIS; S ENTERITIDIS; SALMONELLA TYPHIMURIUM) / (TI;CT;UT)

10	856546 FT=(SHIGELLA; SHIGELLOSIS; BACILLARY DYSENTERY; TOXOPLASMOSIS; TOXOPLASMA; T GONDII; TRICHINOSIS; TRICHINELLA SPIRALIS; T SPIRALIS; YERSINIA; YERSINIOSIS; Y ENTEROCOLITICA; ESCHERICHIA COLI; E COLI; SHIGA TOXIN?; VERO TOXIN?; VTEC; STEC; ENTEROCOCC?) / (TI;CT;UT)

11	58268 FT=(CREUTZFELDT-JAKOB; CREUTZFELDT JAKOB; VCJD; VARIANT CJD; TRANSMISSIBLE SPONGIFORM; LEGIONELLOSIS; LEGIONNAIRES' DISEASE; LEGIONELLA PNEUMOPHILA; L PNEUMOPHILA; SEVERE ACUTE RESPIRATORY SYNDROME; SARS; KLEBSIELLA PLEUMONIAE; K PNEUMOIAE) / (TI;CT;UT)

12	655830 FT=(PSEUDOMONAS AERUGINOSA; PAERUGINOSA; AUREUS; MRSA; STAPHYLOCOCC?; TUBERCULOSIS; TB; MDR TB; MDR-TB; MULTIDRUG-RESISTANT TB) / (TI;CT;UT)

13	89596 FT=(EMERGING INFECTIOUS DISEASE?; INFECTIOUS DISEASE?; COMMUNICABLE DISEASE?; RESPIRATORY VIRUS INFECTIONS; RESPIRATORY VIRAL INFECTIONS; RESPIRATORY INFECTIONS) / (TI;CT;UT)

14	51995 FT=(WATERBORNE # # # # DISEASE?; SOILBORNE # # # # DISEASE?; AIRBORNE # # # # DISEASE?; FOODBORNE # # # # DISEASE?; FOOD INFECTION; FOOD POISONING; SEXUALLY TRANSMITTED DISEASES; STD; SEXUALLY TRANSMITTED AGENTS; EMERGING INFECTIOUS DISEASE?) / (TI;CT;UT)

15	21616 FT=(DIRECT CONTACT TRANSMISSION; PERSON # # PERSON # # # # TRANSMISSION; PERSON # # PERSON # # # # DISEASE?; ANIMAL-TRANSMITTED # # # # DISEASE?; ARTHROPOD-TRANSMITTED # # # # DISEASE?; ZOONOSES; ZOONOSIS) / (TI;CT;UT)

16	5545706 2 TO 15

17	3456 FT=(DISABILITY ADJUSTED; DALY; DALYS; "DALY'S") / (TI;AB;CT;UT)

18	964 16 AND 17

19	499 check duplicates: unique in s=18

20	320 19 AND BASE=ME66

**179 19 NOT 20**
