# Supplementary material for: Use of DALYs in economic analyses on interventions for infectious diseases: a systematic review
Source: Epidemiol Infect. 2014 Dec 12;143(9):1791–802. doi: 10.1017/S0950268814001940 (PMC4456769; doi:10.1017/S0950268814001940)
Supplement: Supplementary file 1 [file S0950268814001940sup.zip › S0950268814001940sup002.doc]

**Supplementary Material**

**Appendix II: Overview of the cost categories considered in the different studies, the chosen perspective according to the authors and according to our own definition**

| **Source:** | **Intervention costs (IC) / program costs** | **Averted cost-of-illness^a^** | | | **Perspective according to** | |
| --- | --- | --- | --- | --- | --- | --- |
|  |  | **DHC** | **DNHC** | **INHC** | **Author** | **Own definition ^b^** |
| Acuna-Villaorduna et al. | Cost for vaccine and administration | Yes | No | No | Healthcare | Third payer |
| Adam et al. | Program costs (resources used) and training of supporting staff; | No | No | No | Economic perspective | Program |
| Akhavan et al. | Investments for buildings, vehicles and equipment (depreciated) ; recurrent costs (salaries and others) and treatment costs (in- and outpatient) | Yes | No | No | *Not specified* | Third payer |
| Akumu et al. | Vaccine costs and syringe prices; additional costs for surveillance and training activities of staff | Yes | No | No | Public health provider | Third payer |
| Aldrigde et al. | Prevention and treatment intervention costs | No | No | No | *Not specified* | Program |
| Alonso González et al. | Personnel and material costs; cost for medication and iron dose. | Yes | Yes | Yes | Both, health provider and sociocultural | Third payer & Societal |
| Altes et al. | Cost for vaccine and administration | Yes | No | No | Healthcare | Third payer |
| Alvis et al. | Personnel and material costs; cost for vaccine dose. | Yes | No | No | Insurer perspective (for costs) and societal perspective (for DALYs & averted cases) | Third payer |
| Atherly et al. | Cost for vaccine and administration, including vaccine wastage | Yes | No | No | Healthcare system perspective | Third payer |
| Babigumira et al. | Costs of vaccines and injection supplies, cold chain storage at the central level, transport from central to district levels, monitoring, surveillance, personnel salaries, social mobilization, training, and capital costs; and household costs of immunization | Yes | No | No | Limited societal perspective | Third payer ^c^ |
| Baltussen et al. | Cost for required resources (e.g. diagnostic tests, drug use, …) | Yes | No | No | Societal perspective | Third payer |
| Baltussen et al. | Cost for required resources at national level (e.g. central planning, training and administration) & at patient-level (e.g. supplies and equipment related to treatments). | Yes | No | No | Societal perspective | Third payer |
| Becker et al. | Cost of insecticide-treated bed nets; transport costs; staff training and staff wages for distribution | No | No | No | Health provider perspective | Program |
| Berry et al. | Cost for vaccine and administration (i.e. cold chain, transportation, personnel time, and stationery cost); including wastage | Yes | No | No | Health provider perspective | Third payer |
| Bishai et al. | Cost for vaccination; outbreak control and surveillance | Yes | No | Yes | Societal perspective | Limited societal |
| Bos and Postma | Cost for vaccination of infants with HIV vaccine and the counseling of mothers | No | No | No | Societal perspective | Program |
| Broughton | Cost of vaccine purchase; additional training; waste disposal and increased use of syringes. | Yes | Yes | Yes | Societal perspective | Societal |
| Buchanan et al. | Policy delivery cost (i.e. drugs and diagnostic tests) | Yes | No | No | Healthcare provider | Third payer |
| Budke et al. | Control costs (i.e. vaccination of goats and sheep (vaccine & administration); anthelmintic prophylaxis of owned dogs and the distribution of baits for stray dogs (baits and administration)) | Yes | No | Yes | *Not specified* | Limited societal ^d^ |
| Butler et al. | Costs of vaccination | Yes | No | Yes | Societal perspective | Limited societal |
| Castillo et al. | Cost of surveying and spraying houses | Yes | No | No | Health service payer | Third payer |
| Clark et al. | Vaccine and application costs (i.e. cost of additional staff, training, cold chain maintenance, storage space and social mobilization) | Yes | Yes | No | *Not specified* | Limited societal |
| Coleman et al. | Cost of the nets, insecticide, staff, sensitization and awareness campaign, transport, other overheads, and community time. | No | No | No | Societal | Program |
| Constenla | Cost for vaccine and application, including wastage | Yes | Yes | Yes | Societal | Societal |
| Constenla et al. | Cost for vaccine and application, including wastage | Yes | No | No | Healthcare payer | Third payer |
| Constenla et al. | Cost for vaccine and application, including wastage | Yes | No | No | Healthcare payer | Third payer |
| Constenla et al. | Cost for vaccine and application, including wastage | Yes | Yes | Yes | Societal | Societal |
| Constenla et al. | Cost for vaccine and application, including wastage | Yes | Yes | Yes | Societal | Societal |
| Constenla et al. | Cost for vaccine and application, including wastage | Yes | Yes | Yes | Healthcare | Third payer ^e^ |
| Conteh et al. | Costs associated with policy change ; community sensitization; behavior change and communication; drug purchase and distribution; administration, training and management | Yes | Yes | Yes | Societal | Societal |
| Cook et al. | Cost for vaccine, delivering and administration; costs incurred by household members to travel to the vaccination outpost and to wait to receive the vaccine | Yes | Yes | Yes | Societal as well as a public sector financial perspective | Third payer & Societal |
| Cook et al. | Cost for vaccine, delivering and administration; costs incurred by household members to travel to the vaccination outpost and to wait to receive the vaccine | Yes | Yes | Yes | Societal perspective | Societal |
| Currie et al. | Costs of diagnosis, costs of (preventive) treatments | Yes | No | No | *Not specified* | Third payer |
| Dabral et al. | Cost for vaccine, wastage, transport cost, handling charges and use of syringes; time cost, travel cost, surveillance cost, campaign cost and cold chain maintenance cost | No | No | No | Provider’s perspective | Program |
| Dandona et al. | Cost for personnel, recurrent goods, recurrent services, capital goods and office space rentals. | No | No | No | *Not specified* | Program |
| De la Hoz et al. | Cost for vaccine and application (i.e. cold chain, storage, transportation) | Yes | Yes | Yes | Societal perspective | Societal |
| Ding et al. | Cost for vaccine and application | Yes | No | No | *Not specified* | Third payer |
| Dowdy et al. | Diagnostic cost and treatment | Yes | No | No | Perspective of the Indian TB control sector | Third payer |
| Dowdy et al. | Diagnostic cost and treatment | Yes | No | No | Perspective of a ministry of health's hypothetical "TB division” | Third payer |
| Dowdy et al. | Diagnostic cost and treatment | Yes | No | No | Perspective of a public-sector TB control program | Third payer |
| Duintjer et al. | Vaccine, application, wastage and organization costs | Yes | No | No | Societal perspective | Third payer |
| Dziekan et al. | Material resources and staff time | No | No | No | *Not specified* | Program |
| Esposito et al. | Vaccine and application costs | Yes | No | No | Health care system perspective | Third payer |
| Fiedler et al. | Total costs of the EOS/EEOS program (i.e. supplies and payments that UNICEF makes to pay for training, per diems and other aspects). Valuation of time of employees devoted to planning, implementing and supervising the program; Costs for time and other inputs donated or provided in-kind by other agencies and individual members of the community | No | No | No | *Not specified* | Program |
| Fischer et al. | Cost for vaccine, application and wastage | Yes | Yes | Yes | Healthcare and societal perspective | Third payer & Societal |
| Gessner et al. | Cost for vaccine and application | Yes | Yes | No | Governmental and societal perspective | Third payer & limited societal |
| Gilson et al. | Cost of intervention (i.e. capital expenditure and annual recurrent expenditure). Capital costs included training costs and investment costs associated with the intervention, (buildings, equipment, materials, and remuneration of trainers and primary-healthcare-workers during training), and initial supervision when establishing the intervention. Annual recurrent expenditures were laboratory costs, transport, drugs, therapeutic and other consumables, central support and supervision costs. | No | No | No | Incremental cost of the intervention | Program |
| Giorgi et al. | Cost for vaccine and application | Yes | No | No | Public health service | Third payer |
| Goldie et al. | Costs for vaccine, wastage, freight and supplies, administration, immunization support and programmatic costs | Yes | No | No | Modified social perspective | Third payer |
| Goldie et al. | Vaccine, application (incl. freight costs …) & wastage *and/o*r screening, diagnosis and treatment costs are categorized into direct medical costs (e.g. staff, supplies, equipment, and specimen transport), women's time costs (time spent traveling, waiting and receiving care), transportation costs and programmatic costs | Yes | Yes | Yes | Social perspective | Societal |
| Goodchild et al. | Expenditures on diagnosis, treatment and costs of other health services | Yes | No | No | Healthcare system provider perspective and control program perspective | Program & Third payer |
| Goodman et al. | Cost for community mobilization; Cost for training and monitoring of shopkeeper (incl. all associated costs such as transport, ….) | No | No | No | Provider perspective | Program |
| Goodman et al. | Costs for insecticide treatment (with/without providing net); or cost for spraying and insecticide; or cost for distributions of chemoprophylaxis (children/pregnant women); or cost for training of providers, education of patients and carers to improve compliance etc. | Yes | No | No | Provider perspective | Program |
| Griekspoor et al. | Cost for drugs and application costs (i.e. staff, office, transport, …) | No | No | No | Program perspective | Program |
| Griffiths et al. | Vaccine cost and application costs | No | No | No | Public health sector | Program |
| Griffiths et al. | Vaccine cost & application (incl. logistic) | Yes | Yes | No | Societal | Limited societal |
| Griffiths et al. | Costs for vaccine, application and logistic | Yes | No | Yes | Societal perspective | Limited societal |
| Griffiths et al. | Costs for vaccine and applications | Yes | Yes | No | Societal perspective | Limited societal |
| Hanson et al. | Project implementation costs, user contributions (payments for nets and insecticide), travel and time costs incurred by users in connection with the purchasing of nets, and in-kind community contributions to the running of the distribution system (annualized capital costs & recurrent cost) | No | No | No | The perspective taken was the project and the community | Program |
| Hogan et al. | Program costs & patients costs | Yes | No | No | *Not specified* | Third payer |
| Hong et al. | Program cost included both institutional (i.e. screening and diagnosis, personnel, transportation, following-up and supervision) and individual expenses (i.e. medical expenses; lost income due work absence; travel costs). | Yes | Yes | Yes | *Not specified* | Societal |
| Hutton et al. | Costs for planning and management, sensitization, drugs, drug distribution, … | Yes | No | No | *Not specified* | Third payer |
| Jeuland et al. | Vaccine manufacturing and delivery costs | Yes | Yes | Yes | Public sector financial perspective (& in supplementary social) | Third payer & Societal |
| John et al. | Personnel costs for nurse counselors and lab technicians, and the time taken for group information, pre- and post-test counseling. | No | No | No | *Not specified* | Program |
| Khan et al. | Cost for vaccine and application | Yes | No | No | *Not specified* | Third payer |
| Kim et al. | Costs for vaccine, application and wastage | Yes | Yes | Yes | Societal perspective | Societal |
| Kim et al. | Costs for vaccine, application and wastage | Yes | Yes | Yes | Societal and payers perspective | Third payer & Societal |
| Kim et al. | Costs for vaccine, application and wastage | Yes | Yes | Yes | Societal and payers perspective | Third payer & Societal |
| Kim et al. | Costs for vaccine, application and wastage (including surveillance, monitoring and social mobilization). | Yes | No | No | Limited societal perspective | Third payer |
| Lee et al. | Costs for vaccine | Yes | No | Yes | Societal perspective | Limited societal |
| Levin et al. | Cost for personnel, vaccines, injection supplies, transportation and social mobilization. Further societal costs (i.e. transportation, travel, and waiting time) for obtaining measles vaccinations. | Yes | No | No | *Not specified* | Third payer ^c^ |
| Lima et al. | Direct costs based on treating an individual with first-line therapy during a full year. | Yes | No | No | *Not specified* | Third payer |
| Liu et al. | Vaccine, application (incl. logistic) and wastage | Yes | Yes | Yes | Health care system perspective & societal perspective | Third payer & Societal |
| Lubell et al. | Treatment cost | Yes | Yes | Yes | Provider perspective & societal perspective | Third payer & Societal |
| Lubell et al. | Treatments, diagnostic tests and medical equipment; hotel costs of inpatient care; | Yes | No | No | Provider perspective (health facilities) | Third payer |
| Lutumba et al. | Comparing two alternatives | Yes | Yes | Yes | *Not specified* | Societal |
| Luz et al. | Costs for larval control programs | Yes | Yes | Yes | Societal perspective | Societal |
| Maleewong et al. | Comparing two alternatives | Yes | No | No | Healthcare provider | Third payer |
| Mangen et al. | Intervention costs in slaughterhouse (annualized capital costs & recurrent costs) | Yes | Yes | Yes | Societal perspective | Societal |
| Mangen et al. | Intervention costs at farm-level and/or slaughterhouse (annualized capital costs & recurrent costs) | Yes | Yes | Yes | Societal perspective | Societal |
| Mangen et al. | Vaccine costs, application & wastage | Yes | Yes | Yes | Third payer and societal | Third payer & Societal |
| Marseille et al. | Program costs (counseling, testing and drug acquisition) | Yes | No | No | Medical system perspective | Third payer |
| Marseille et al. | Program costs (counseling, testing and drug acquisition) | Yes | No | No | Public sector health payer | Third payer |
| Marseille et al. | Costs of the drugs and the cost of patient monitoring | Yes | No | Yes | Company’s perspective | Provider perspective ^i^ |
| Marseille et al. | Comparing two alternatives | Yes | No | No | Societal perspective | Third payer |
| Mbonye et al. | Program costs (i.e. drug costs, recurrent and capital costs) | Yes | Yes | Yes | *Not specified* | Societal |
| Meya et al. | Total costs encompassing reagents, daily positive-negative quality controls, laboratory disposable supplies, labor, external quality assurance testing, laboratory overhead and margin | No | No | No | *Not specified* | Program |
| Miller et al. | Vaccine cost (doses delivered plus 10% wastage), syringe cost, administration cost and the cost associated with adverse reactions | Yes | No | No | Public healthcare payer perspective | Third payer |
| Mogasale & Barendregt | Vaccine cost, application and wastage | Yes | No | No | Healthcare payer | Third payer |
| Mohan et al. | Compare two cohorts | Yes | Yes | Yes | Societal perspective | Societal |
| Morel et al. | Costs for resources needed to provide the intervention | No | No | No | *Not specified* | Program |
| Mueller et al. | Annualized capital and recurrent costs required in the delivery of the campaign (i.e. personnel, overheads, and transport) | Yes | No | No | Provider perspective, only the costs and effects borne by the ministry of health and donors are considered | Program & Third payer |
| Muennig et al. | Drug costs plus pharmacy fee | Yes | No | Yes | Societal perspective | Societal |
| Murray et al. | Vaccine costs and application / costs of water and sanitation programs | Yes | No | No | Public health decision-makers | Third payer |
| Navas et al. | Vaccine costs & application | Yes | Yes | Yes | Provider's perspective & societal | Third payer & Societal |
| Ono et al. | Immunization and treatment costs | Yes | Yes | No | Medical service decision-maker who cares about both public and private costs | Limited societal |
| Orlando et al. | Program costs as observed in an implemented program | Yes | No | No | Private perspective & public perspective | Program & Third payer |
| Ortega et al. | Vaccine costs & application | Yes | No | No | Governmental perspective | Third payer |
| Pitter et al. | Program costs (medication & distribution) | Yes | No | No | Perspective of public sector health payer | Third payer |
| Platonov et al. | Vaccine costs & application | Yes. | No | Yes | Societal perspective | Societal |
| Politi et al. | Cost for drugs and treatment of side-effects | Yes | No | Yes | Societal perspective | Limited societal |
| Prakash et al. | Costs for vaccine and administration (i.e. vaccine storage, vaccine transportation, personnel, supervision, and space) | Yes | No | No | Societal perspective | Third payer |
| Prinja et al. | Costs for implementing targeted interventions | Yes | No | No | Health system perspective; | Third payer |
| Reithinger et al. | Non-medical costs (staff, tools etc.) and medical costs (medication etc.) & overhead | Yes | No | No | *Not specified* | Third payer |
| Renaud et al. | Package costs (outpatient visit; laboratory; voluntary counseling and testing; adherence counseling; psychological and social support; food support) | Yes | No | No | Healthcare provider perspective | Program |
| Rheingans et al. | Vaccine cost, application & wastage | Yes | Yes | Yes | Healthcare payer perspective | Third payer ^g^ |
| Rheingans et al. | Vaccine cost, application & wastage | Yes | Yes | Yes | Healthcare payer perspective | Third payer ^g^ |
| Robberstad et al. | Program costs, including capital and recurrent cost | Yes | No | No | Perspective of the local health care provider | Third payer |
| Ross et al. | Drug cost, drug dispensing and supplies, delivery of drugs, supervision, training and sensitization | Yes | Yes | No | Not specified | Limited societal |
| Roth et al. | Vaccine costs, service costs of vaccination (transportation, cold chain, and veterinary fees); costs related to ear tagging; service costs for surveillance and diagnostic tests; and costs of health education, training, and advocacy for herders. | Yes | Yes | Yes | Public health sector perspective and societal perspective ^d^ | Third payer ^h^ & societal ^d, h^ |
| Ryan et al. | Drug costs etc. | Yes | No | No | Health service perspective | Third payer |
| Schackman et al. | Laboratory costs, testing costs (test & application) and treatment costs. But also patient time for treatment and follow-up visits | Yes | Yes | Yes | Societal perspective | Societal |
| Scuffham & McIntyre | Costs for vaccine and application | Yes | No | No | The cost to the Australian health system | Third payer |
| Shah et al. | Costs for preventive treatment, application and diagnostic | Yes | No | No | Health-system perspective | Third payer |
| Shaw & Cattand | Costs related to screening and preventive treatment (e.g. drugs) | Yes | No | No | Point of view of those allocating funds within the health sector | Third payer |
| Shepard et al. | Costs for vaccine and application | Yes | Yes | Yes | Societal perspective | Societal |
| Shillcut et al. | Diagnostic and treatment cost | Yes | Yes | No | Joint perspective of providers and patients | Limited societal |
| Shrestha et al. | Capital and recurrent costs of intervention | Yes | No | No | Perspective of the health care payer who would finance the intervention | Third payer |
| Sicuri et al. | Drug costs | Yes | Yes | Yes | Health system and households | Societal |
| Sinha et al. | Costs for vaccine and administration | Yes | Yes | Yes | Societal perspective | Societal |
| Sinha et al. | Costs for vaccine and administration | Yes | Yes | Yes | Healthcare payer perspective and societal perspective | Third payer & Societal |
| Skull & Butler | Costs for vaccine, administration, treatment of side-effects, campaign promotion | Yes | No | Yes | Societal perspective | Program & third payer ^n^ |
| Skull et al. | Costs for vaccine, administration, treatment of side-effects, campaign promotion | Yes | No | Yes | Societal perspective | Program & third payer ^n^ |
| Suarez et al. | Cost of drugs and other cost per patient treated | Yes | No | No | Perspective of  the public sector | Third payer |
| Suaya et al. | Operational (i.e. pre-packing the larvicide, training the field workers, transporting and applying the larvicide, and supervision) and administration costs as well as annual media and communications campaigns | Yes | Yes | Yes | Both, public sector and societal perspective | Third payer & Societal |
| Sweat et al. | Pre-client quantity of goods and services used in delivery of the intervention. | No | No | No | Program perspective | Program |
| Sweat et al. | Enhancements to the health system to enable the intervention; the cost of pre-test counseling; the cost of HIV testing and post-test counseling; cost of nevirapine for mother and infant | Yes | No | No | Program perspective | Program & Third payer |
| Sweat et al. | Pre-client quantity of goods and services used in delivery of the intervention | No | No | No | Perspective of implementing NGOs | Program |
| Tate et al. | Costs for vaccine, application and waste | Yes | Yes | Yes | *Not specified* | Societal |
| Terris-Presholt et al. | All resources used in the intervention (capital, including training; personal, supplies, transportation, …) | No | No | No | Providers’ perspective | Program |
| Thielman et al. | Testing costs (personal, tests and laboratory consumables) and overhead (e.g. building costs, telephone etc.) and costs for antiretroviral therapy and costs for tuberculosis prophylaxis | No | No | No | Health policy point of view | Program |
| Torgerson et al. | Comparing two alternatives | Yes | No | Yes | *Not specified* | Limited societal |
| Touch et al. | Costs for vaccine, application, wastage and operational costs, as well as introduction costs (e.g. training and information, education and communication materials) | Yes | Yes | Yes | Both provider and societal perspective | Third payer & Societal |
| Tozan et al. | Patient-related costs (i.e. drug, time spend by community healthcare worker) and program-related costs | Yes | No | No | Provider perspective | Third payer |
| Tupasi et al. | Direct costs of interventions (drugs, visits) and programme related costs (Vehicle and equipment costs, training of healthcare workers, building costs). | Yes | No | No | Societal perspective | Third payer |
| Uruena et al. | Vaccine costs, including wastage, expansion of cold chain, transportation, materials, training, supervision and monitoring. | Yes | Yes | Yes | Health care provider & societal perspective | Third payer & Societal |
| Uthman et al. | The cost of intervention and complications of the intervention. | Yes | No | No | Government health care payer | Third payer |
| Utzinger et al. | Interventions costs (i.e. personal and material costs) and annual maintenance costs | No ^j^ | Yes | Yes | Company perspective | Societal ^j^ |
| Utzinger et al. | Interventions costs (i.e. personal and material costs) and annual maintenance costs | No | No | No | *Not specified* | Program |
| Van Hulst et al. | Screening costs. | Yes | No | Yes | Societal perspective | Limited societal |
| Varley et al. | Costs for water sanitation and educational program. | No | No | No | *Not specified* | Program |
| Vega et al. | Treatment costs (both medical and non-medical) | Yes | No | No | *Not specified* | Third payer |
| Vespa et al. | Vaccination costs (incl. administration, vaccine costs, expected losses from waste). | Yes | Yes | Yes | Societal perspective (base case scenario) | Societal |
| Vickerman et al. Tanzania | Costs of screening tests | Yes | No | No | *Not specified* | Third payer |
| Vickerman et al. South Africa | Intervention costs (drugs, health care workers) | No | No | No | Provider perspective | Program |
| Wang et al. | Program development and update, training of counsellors, counselling and testing, increased condom use among clients, administration, and evaluation. | Yes | No | No | Societal perspective | Third payer |
| Wilkinson et al. | Drug costs, HIV test costs, the cost of extra staff, training and health education. | No | No | No | Health system approach | Program |
| Wilopo et al. | Vaccination costs (administration, vaccine, and wastage) | Yes | Yes | Yes | Societal & healthcare system perspective | Third payer & Societal |
| Wu et al. | Expenses for drugs, chest X-ray films, sputum smear and cultures for the patients. | No | No | No | *Not specified* | Program |
| Xu et al. | Expenses for drugs, chest X-ray films, sputum smear and cultures for the patients. | Yes | No | No | *Not specified* | Third payer |
| Xu et al. | Expenses for drugs, chest X-ray films, sputum smear and cultures for the patients. | Yes | No | No | *Not specified* | Third payer |
| Yukich et al. | Capital and recurrent costs, incl. commodities and their delivery, health promotion, and social mobilization, training of health workers, health promotion and social mobilization, use of facilities, transportation, payment of personnel, and the commodities themselves. | No | No | No | Provider perspective | Program |
| Zhang et al. | Distributing syringes, over all necessary infrastructure, personnel, marketing and recurring service costs. | Yes | No | No | Governmental and societal perspective | Third payer |
| Zinsstag et al. | Dog-vaccination campaign | No | No | No | Policy-oriented perspective | Program |

**Note:**

a) Averted cost-of-illness are: i) direct healthcare costs (DHC) such as costs for medical services provided by GPs and specialists, medication, hospitalization, etc.; ii) direct non-healthcare costs (DNHC) such as travel costs and other costs paid by patients themselves; and iii) indirect non-healthcare costs (INHC), such as productivity losses of patients and/or caregivers being absent from paid work, special education, etc..
b) For the definition used see Table 2.
c) Broader perspective for intervention costs (e.g. also considering the household costs) than for averted cost-of-illness where the only category considered was DHC.
d) In addition to the averted cost-of-illness related to human health, the impact of the intervention on the agriculture sector (i.e. averted livestock losses) was also taken into consideration in this economic evaluation.
e) Although DNHC and INHC were described in the methodology, the economic evaluation was only applied using a third-payer perspective.
f) In addition to the intervention costs, HIV-related costs for the employer were also considered, those being: Net present value of medical care costs, HIV-related productivity and training losses, disability payments, and death and funeral benefits.
g) The authors used the third-payer perspective whereby only averted DHC was taken into consideration; however, they also presented the break-even point using both a medical and societal perspective. For the latter averted DHC, DNHC and productivity losses were considered.
h) The program costs were paid by the Ministry of Agriculture, therefore “third payer” is in this study equivalent to “ministry of agriculture”.
i) Cost-effectiveness ratios were presented using a program and a third-payer perspective. INHC were considered in the presented cost-benefit ratios.
j) Authors assumed that treatment costs and productivity losses due to absenteeism were borne by mine employees (and therefore considered as costs paid by the patients themselves). Whereas the intervention costs would be funded by the company.

**References:**

1.	**Acuna-Villaorduna C *et al***. Cost-effectiveness analysis of introduction of rapid, alternative methods to identify multidrug-resistant tuberculosis in middle-income countries. *Clinical Infectious Diseases* 2008, **47**:487-495.

2.	**Adam T *et al***. Cost effectiveness analysis of strategies for maternal and neonatal health in developing countries. *British Medical Journal* 2005, **331**:1107.

3.	**Akhavan D *et al***. Cost-effective malaria control in Brazil. Cost-effectiveness of a Malaria Control Program in the Amazon Basin of Brazil, 1988-1996. *Social Science & Medicine* 1999, **49**:1385-1399.

4.	**Akumu AO *et al***. Economic evaluation of delivering Haemophilus influenzae type b vaccine in routine immunization services in Kenya. *Bulletin of the World Health Organization* 2007, **85**:511-518.

5.	**Aldridge RW *et al***. Determining a cost effective intervention response to HIV/AIDS in Peru. *BMC Public Health* 2009, **9**:352.

6.	**Alonso PL *et al***. Cost-effectiveness of iron supplementation and malaria chemoprophylaxis in the prevention of anaemia and malaria among Tanzanian infants. *Bulletin of the World Health Organization* 2000, **78**:97-107.

7.	**Altes HK *et al***. Targeted BCG vaccination against severe tuberculosis in low-prevalence settings: epidemiologic and economic assessment. *Epidemiology* 2009, **20**:562-568.

8.	**Alvis N *et al***. [Economic impact of introducing the injectable inactivated polio vaccine in Colombia]. *Revista Panamericana de Salud Pública* 2010, **27**:352-359.

9.	**Atherly D *et al***. Rotavirus vaccination: cost-effectiveness and impact on child mortality in developing countries. *Journal of Infectious Diseases* 2009, **200 Suppl 1**:S28-38.

10.	**Babigumira JB *et al***. Assessing the cost-effectiveness of measles elimination in Uganda: local impact of a global eradication program. *Journal of Infectious Diseases* 2011, **204 Suppl 1**:S116-123.

11.	**Baltussen R *et al***. Cost effectiveness analysis of strategies for tuberculosis control in developing countries. *British Medical Journal* 2005, **331**:1364.

12.	**Baltussen RM *et al***. Cost-effectiveness of trachoma control in seven world regions. *Ophthalmic Epidemiology* 2005, **12**:91-101.

13.	**Becker-Dreps SI *et al***. Cost-effectiveness of adding bed net distribution for malaria prevention to antenatal services in Kinshasa, Democratic Republic of the Congo. *American Journal of Tropical Medicine and Hygiene* 2009, **81**:496-502.

14.	**Berry SA *et al***. The cost-effectiveness of rotavirus vaccination in Malawi. *Journal of Infectious Diseases* 2010, **202 Suppl**:S108-115.

15.	**Bishai D *et al***. The cost-effectiveness of supplementary immunization activities for measles: a stochastic model for Uganda. *Journal of Infectious Diseases* 2011, **204 Suppl 1**:S107-115.

16.	**Bos JM, Postma MJ**. The economics of HIV vaccines: projecting the impact of HIV vaccination of infants in sub-Saharan Africa. *Pharmacoeconomics* 2001, **19**:937-946.

17.	**Broughton EI**. Economic evaluation of Haemophilus influenzae type B vaccination in Indonesia: a cost-effectiveness analysis. *Journal of Public Health* 2007, **29**:441-448.

18.	**Buchanan J *et al***. Cost-effectiveness of pre-referral antimalarial, antibacterial, and combined rectal formulations for severe febrile illness. *PLoS ONE* 2010, **5**:e14446.

19.	**Budke CM *et al***. Economic effects of echinococcosis in a disease-endemic region of the Tibetan Plateau. *American Journal of Tropical Medicine and Hygiene* 2005, **73**:2-10.

20.	**Butler JR *et al***. The cost-effectiveness of pneumococcal conjugate vaccination in Australia. *Vaccine* 2004, **22**:1138-1149.

21.	**Castillo-Riquelme M *et al***. Modelling geographic variation in the cost-effectiveness of control policies for infectious vector diseases: the example of Chagas disease. *Journal of Health Economics* 2008, **27**:405-426.

22.	**Clark AD *et al***. Cost-effectiveness of rotavirus vaccination in peru. *Journal of Infectious Diseases* 2009, **200 Suppl 1**:S114-124.

23.	**Coleman PG *et al***. Rebound mortality and the cost-effectiveness of malaria control: potential impact of increased mortality in late childhood following the introduction of insecticide treated nets. *Tropical Medicine & International Health* 1999, **4**:175-186.

24.	**Constenla DO**. Economic impact of pneumococcal conjugate vaccination in Brazil, Chile, and Uruguay. *Revista Panamericana de Salud Pública* 2008, **24**:101-112.

25.	**Constenla D *et al***. [Potential cost effectiveness of a rotavirus vaccine in Chile]. *Revista Médica de Chile* 2006, **134**:679-688.

26.	**Constenla D *et al***. Economic impact of a rotavirus vaccination program in Mexico. *Revista Panamericana de Salud Pública* 2009, **25**:481-490.

27.	**Constenla D *et al***. [Assessment of the economic impact of the antiretroviral vaccine in Venezuela]. *Revista Panamericana de Salud Pública* 2006, **20**:213-222.

28.	**Constenla D *et al***. [Economic impact of rotavirus vaccination in Panama]. *Anales de pediatría* 2008, **68**:128-135.

29.	**Constenla DO *et al***. Economic impact of a rotavirus vaccine in Brazil. *Journal of Health, Population and Nutrition* 2008, **26**:388-396.

30.	**Conteh L *et al***. The cost-effectiveness of intermittent preventive treatment for malaria in infants in Sub-Saharan Africa. *PLoS ONE* 2010, **5**:e10313.

31.	**Cook J *et al***. The cost-effectiveness of typhoid Vi vaccination programs: calculations for four urban sites in four Asian countries. *Vaccine* 2008, **26**:6305-6316.

32.	**Cook J *et al***. Evaluating investments in typhoid vaccines in two slums in Kolkata, India. *Journal of Health, Population and Nutrition* 2009, **27**:711-724.

33.	**Currie CS *et al***. Cost, affordability and cost-effectiveness of strategies to control tuberculosis in countries with high HIV prevalence. *BMC Public Health* 2005, **5**:130.

34.	**Dabral M**. Cost-effectiveness of supplementary immunization for measles in India. *Indian Pediatrics* 2009, **46**:957-962.

35.	**Dandona L *et al***. Cost-effectiveness of HIV prevention interventions in Andhra Pradesh state of India. *BMC Health Services Research* 2010, **10**:117.

36.	**De la Hoz F *et al***. Potential epidemiological and economical impact of two rotavirus vaccines in Colombia. *Vaccine* 2010, **28**:3856-3864.

37.	**Ding D *et al***. Cost-effectiveness of routine immunization to control Japanese encephalitis in Shanghai, China. *Bulletin of the World Health Organization* 2003, **81**:334-342.

38.	**Dowdy DW *et al***. Serological testing versus other strategies for diagnosis of active tuberculosis in India: a cost-effectiveness analysis. *PLoS Medicine* 2011, **8**:e1001074.

39.	**Dowdy DW *et al***. Cost-effectiveness of novel diagnostic tools for the diagnosis of tuberculosis. *International Journal of Tuberculosis and Lung Disease* 2008, **12**:1021-1029.

40.	**Dowdy DW *et al***. Impact and cost-effectiveness of culture for diagnosis of tuberculosis in HIV-infected Brazilian adults. *PLoS ONE* 2008, **3**:e4057.

41.	**Duintjer Tebbens RJ *et al***. Economic analysis of the global polio eradication initiative. *Vaccine* 2010, **29**:334-343.

42.	**Dziekan G *et al***. The cost-effectiveness of policies for the safe and appropriate use of injection in healthcare settings. *Bulletin of the World Health Organization* 2003, **81**:277-285.

43.	**Esposito DH *et al***. Projected impact and cost-effectiveness of a rotavirus vaccination program in India, 2008. *Clinical Infectious Diseases* 2011, **52**:171-177.

44.	**Fiedler JL, Chuko T**. The cost of Child Health Days: a case study of Ethiopia's Enhanced Outreach Strategy (EOS). *Health Policy and Planning* 2008, **23**:222-233.

45.	**Fischer TK *et al***. Health care costs of diarrheal disease and estimates of the cost-effectiveness of rotavirus vaccination in Vietnam. *Journal of Infectious Diseases* 2005, **192**:1720-1726.

46.	**Gessner BD *et al***. Vaccine-preventable haemophilus influenza type B disease burden and cost-effectiveness of infant vaccination in Indonesia. *Pediatric Infectious Disease Journal* 2008, **27**:438-443.

47.	**Gilson L *et al***. Cost-effectiveness of improved treatment services for sexually transmitted diseases in preventing HIV-1 infection in Mwanza Region, Tanzania. *Lancet* 1997, **350**:1805-1809.

48.	**Giorgi-Rossi P *et al***. Cost-effectiveness of introducing the conjugated pneumococcal vaccine to routine free immunizations for infants in Lazio, Italy. *Health Policy* 2009, **89**:225-238.

49.	**Goldie SJ *et al***. Health and economic outcomes of HPV 16,18 vaccination in 72 GAVI-eligible countries. *Vaccine* 2008, **26**:4080-4093.

50.	**Goldie SJ *et al***. Mathematical models of cervical cancer prevention in the Asia Pacific region. *Vaccine* 2008, **26 Suppl 12**:M17-29.

51.	**Goodchild M *et al***. A cost-benefit analysis of scaling up tuberculosis control in India. *International Journal of Tuberculosis and Lung Disease* 2011, **15**:358-362.

52.	**Goodman CA *et al***. The cost-effectiveness of improving malaria home management: shopkeeper training in rural Kenya. *Health Policy and Planning* 2006, **21**:275-288.

53.	**Goodman CA *et al***. Cost-effectiveness of malaria control in sub-Saharan Africa. *The Lancet* 1999, **354**:378-385.

54.	**Griekspoor A *et al***. Cost-effectiveness analysis of humanitarian relief interventions: visceral leishmaniasis treatment in the Sudan. *Health Policy and Planning* 1999, **14**:70-76.

55.	**Griffiths UK *et al***. Incremental cost-effectiveness of supplementary immunization activities to prevent neonatal tetanus in Pakistan. *Bulletin of the World Health Organization* 2004, **82**:643-651.

56.	**Griffiths UK *et al***. The cost-effectiveness of introducing hepatitis B vaccine into infant immunization services in Mozambique. *Health Policy and Planning* 2005, **20**:50-59.

57.	**Griffiths UK *et al***. The cost-effectiveness of alternative polio immunization policies in South Africa. *Vaccine* 2006, **24**:5670-5678.

58.	**Griffiths UK *et al***. Comparative economic evaluation of Haemophilus influenzae type b vaccination in Belarus and Uzbekistan. *PLoS ONE* 2011, **6**:e21472.

59.	**Hanson K *et al***. Cost-effectiveness of social marketing of insecticide-treated nets for malaria control in the United Republic of Tanzania. *Bulletin of the World Health Organization* 2003, **81**:269-276.

60.	**Hogan DR *et al***. Cost effectiveness analysis of strategies to combat HIV/AIDS in developing countries. *British Medical Journal* 2005, **331**:1431-1437.

61.	**Hong FC *et al***. Congenital syphilis: an economic evaluation of a prevention program in China. *Sexually Transmitted Diseases* 2010, **37**:26-31.

62.	**Hutton G *et al***. Cost-effectiveness of malaria intermittent preventive treatment in infants (IPTi) in Mozambique and the United Republic of Tanzania. *Bulletin of the World Health Organization* 2009, **87**:123-129.

63.	**Jeuland M *et al***. Cost-effectiveness of new-generation oral cholera vaccines: a multisite analysis. *Value Health* 2009, **12**:899-908.

64.	**John FN *et al***. Cost effectiveness of couple counselling to enhance infant HIV-1 prevention. *International Journal of STD & AIDS* 2008, **19**:406-409.

65.	**Khan MM, Ehreth J**. Costs and benefits of polio eradication: a long-run global perspective. *Vaccine* 2003, **21**:702-705.

66.	**Kim SY *et al***. Economic evaluation of pneumococcal conjugate vaccination in The Gambia. *BMC Infectious Diseases* 2010, **10**:260.

67.	**Kim SY *et al***. Economic evaluation of hepatitis B vaccination in low-income countries: using cost-effectiveness affordability curves. *Bulletin of the World Health Organization* 2007, **85**:833-842.

68.	**Kim SY *et al***. Cost-effectiveness of Rotavirus vaccination in Vietnam. *BMC Public Health* 2009, **9**:29.

69.	**Kim SY *et al***. Comparative evaluation of the potential impact of rotavirus versus HPV vaccination in GAVI-eligible countries: a preliminary analysis focused on the relative disease burden. *BMC Infectious Diseases* 2011, **11**:174.

70.	**Lee BY *et al***. The potential economic value of a Trypanosoma cruzi (Chagas disease) vaccine in Latin America. *PLoS Neglected Tropical Diseases* 2010, **4**:e916.

71.	**Levin A *et al***. Global eradication of measles: an epidemiologic and economic evaluation. *Journal of Infectious Diseases* 2011, **204 Suppl 1**:S98-106.

72.	**Lima VD *et al***. Expanding HAART treatment to all currently eligible individuals under the 2008 IAS-USA Guidelines in British Columbia, Canada. *PLoS ONE* 2010, **5**:e10991.

73.	**Liu W *et al***. Cost-effectiveness of Japanese encephalitis (JE) immunization in Bali, Indonesia. *Vaccine* 2008, **26**:4456-4460.

74.	**Lubell Y *et al***. An economic evaluation of home management of malaria in Uganda: an interactive Markov model. *PLoS ONE* 2010, **5**:e12439.

75.	**Lubell Y *et al***. Cost-effectiveness of parenteral artesunate for treating children with severe malaria in sub-Saharan Africa. *Bulletin of the World Health Organization* 2011, **89**:504-512.

76.	**Lutumba P *et al***. Human African trypanosomiasis in a rural community, Democratic Republic of Congo. *Emerging Infectious Diseases* 2007, **13**:248-254.

77.	**Luz PM *et al***. Dengue vector control strategies in an urban setting: an economic modelling assessment. *Lancet* 2011, **377**:1673-1680.

78.	**Maleewong U *et al***. The cost-effectiveness analysis of initiating HIV/AIDS treatment with efavirenz-based regimens compared with nevirapine-based regimens in Thailand. *Journal of the Medical Association of Thailand* 2008, **91 Suppl 2**:S126-138.

79.	**Mangen MJJ *et al***. Economic analysis of Campylobacter control in the Dutch broiler meat chain. *Agribusiness* 2007, **23**:173-192.

80.	**Mangen MJ *et al***. Cost-utility analysis to control Campylobacter on chicken meat: dealing with data limitations. *Risk Analysis* 2007, **27**:815-830.

81.	**Mangen M-JM *et al***. Is it cost-effective to introduce rotavirus vaccination in the Dutch national immunization program? *Vaccine* 2010, **28**:2624-2635.

82.	**Marseille E *et al***. Cost-effectiveness of antiviral drug therapy to reduce mother-to-child HIV transmission in sub-Saharan Africa. *AIDS* 1998, **12**:939-948.

83.	**Marseille E *et al***. Cost effectiveness of single-dose nevirapine regimen for mothers and babies to decrease vertical HIV-1 transmission in sub-Saharan Africa. *The Lancet* 1999, **354**:803-809.

84.	**Marseille E *et al***. The costs and benefits of private sector provision of treatment to HIV-infected employees in Kampala, Uganda. *AIDS* 2006, **20**:907-914.

85.	**Marseille E *et al***. The cost effectiveness of home-based provision of antiretroviral therapy in rural Uganda. *Applied Health Economics and Health Policy* 2009, **7**:229-243.

86.	**Mbonye AK *et al***. Intermittent preventive treatment of malaria in pregnancy: the incremental cost-effectiveness of a new delivery system in Uganda. *Transactions of the Royal Society of Tropical Medicine and Hygiene* 2008, **102**:685-693.

87.	**Meya DB *et al***. Cost-effectiveness of serum cryptococcal antigen screening to prevent deaths among HIV-infected persons with a CD4+ cell count < or = 100 cells/microL who start HIV therapy in resource-limited settings. *Clinical Infectious Diseases* 2010, **51**:448-455.

88.	**Miller MA**. An assessment of the value of Haemophilus influenzae type b conjugate vaccine in Asia. *Pediatric Infectious Disease Journal* 1998, **17**:S152-159.

89.	**Mogasale V, Barendregt J**. Cost-effectiveness of influenza vaccination of people aged 50-64 years in Australia: results are inconclusive. *Australian and New Zealand Journal of Public Health* 2011, **35**:180-186.

90.	**Mohan CI *et al***. The cost-effectiveness of DOTS in urban Brazil. *International Journal of Tuberculosis and Lung Disease* 2007, **11**:27-32.

91.	**Morel CM *et al***. Cost effectiveness analysis of strategies to combat malaria in developing countries.[Erratum appears in BMJ. 2006 Jan 21;332(7534):151].[Erratum appears in BMJ. 2006 Jul 8;333(7558):86]. *British Medical Journal* 2005, **331**:1299.

92.	**Mueller DH *et al***. Cost-effectiveness analysis of insecticide-treated net distribution as part of the Togo Integrated Child Health Campaign. *Malaria Journal* 2008, **7**:73.

93.	**Muennig P *et al***. The cost effectiveness of strategies for the treatment of intestinal parasites in immigrants. *New England Journal of Medicine* 1999, **340**:773-779.

94.	**Murray J *et al***. Cost-effectiveness of oral cholera vaccine in a stable refugee population at risk for epidemic cholera and in a population with endemic cholera. *Bulletin of the World Health Organization* 1998, **76**:343-352.

95.	**Navas E *et al***. Cost-benefit and cost-effectiveness of the incorporation of the pneumococcal 7-valent conjugated vaccine in the routine vaccination schedule of Catalonia (Spain). *Vaccine* 2005, **23**:2342-2348.

96.	**Ono S *et al***. Cost-effectiveness analysis of antiretroviral drug treatment and HIV-1 vaccination in Thailand. *Japanese journal of infectious diseases* 2006, **59**:168-173.

97.	**Orlando S *et al***. Cost-Effectiveness of Using HAART in Prevention of Mother-To-Child Transmission in the DREAM-Project Malawi. *Journal of Acquired Immune Deficiency Syndromes* 2010, **55**:631-634.

98.	**Ortega O *et al***. Cost-benefit analysis of a rotavirus immunization program in the Arab Republic of Egypt. *Journal of Infectious Diseases* 2009, **200 Suppl 1**:S92-98.

99.	**Pitter C *et al***. Cost-effectiveness of cotrimoxazole prophylaxis among persons with HIV in Uganda. *Journal of Acquired Immune Deficiency Syndromes* 2007, **44**:336-343.

100.	**Platonov AE *et al***. Economic evaluation of Haemophilus influenzae type b vaccination in Moscow, Russian Federation. *Vaccine* 2006, **24**:2367-2376.

101.	**Politi C *et al***. Cost-effectiveness analysis of alternative treatments of African gambiense trypanosomiasis in Uganda. *Health Econ* 1995, **4**:273-287.

102.	**Prakash C**. Crucial factors that influence cost-effectiveness of universal hepatitis B immunization in India. *International Journal of Technology Assessment in Health Care* 2003, **19**:28-40.

103.	**Prinja S *et al***. Cost effectiveness of targeted HIV prevention interventions for female sex workers in India. *Sexually Transmitted Infections* 2011, **87**:354-361.

104.	**Reithinger R, Coleman PG**. Treating cutaneous leishmaniasis patients in Kabul, Afghanistan: cost-effectiveness of an operational program in a complex emergency setting. *BMC Infectious Diseases* 2007, **7**:3.

105.	**Renaud A *et al***. The cost effectiveness of integrated care for people living with HIV including antiretroviral treatment in a primary health care centre in Bujumbura, Burundi. *AIDS Care* 2009, **21**:1388-1394.

106.	**Rheingans RD *et al***. Potential cost-effectiveness of vaccination for rotavirus gastroenteritis in eight Latin American and Caribbean countries. *Revista Panamericana de Salud Pública* 2007, **21**:205-216.

107.	**Tate JE *et al***. Rotavirus disease burden and impact and cost-effectiveness of a rotavirus vaccination program in kenya. *Journal of Infectious Diseases* 2009, **200 Suppl 1**:S76-84.

108.	**Robberstad B, Evjen-Olsen B**. Preventing mother to child transmission of HIV with highly active antiretroviral treatment in Tanzania--a prospective cost-effectiveness study. *Journal of Acquired Immune Deficiency Syndromes* 2010, **55**:397-403.

109.	**Ross A *et al***. Determinants of the cost-effectiveness of intermittent preventive treatment for malaria in infants and children. *PLoS ONE* 2011, **6**:e18391.

110.	**Roth F *et al***. Human health benefits from livestock vaccination for brucellosis: case study. *Bulletin of the World Health Organization* 2003, **81**:867-876.

111.	**Ryan M *et al***. The cost-effectiveness of cotrimoxazole prophylaxis in HIV-infected children in Zambia. *AIDS* 2008, **22**:749-757.

112.	**Schackman BR *et al***. Cost-effectiveness of rapid syphilis screening in prenatal HIV testing programs in Haiti. *PLoS Med* 2007, **4**:e183.

113.	**Scuffham PA, McIntyre PB**. Pertussis vaccination strategies for neonates--an exploratory cost-effectiveness analysis. *Vaccine* 2004, **22**:2953-2964.

114.	**Shah M *et al***. Cost-effectiveness of new WHO recommendations for prevention of mother-to-child transmission of HIV in a resource-limited setting. *AIDS* 2011, **25**:1093-1102.

115.	**Shaw AP, Cattand P**. Analytical tools for planning cost-effective surveillance in Gambiense sleeping sickness. *Medecine tropicale* 2001, **61**:412-421.

116.	**Shepard DS *et al***. Cost-effectiveness of a pediatric dengue vaccine. *Vaccine* 2004, **22**:1275-1280.

117.	**Shillcutt S *et al***. Cost-effectiveness of malaria diagnostic methods in sub-Saharan Africa in an era of combination therapy. *Bulletin of the World Health Organization* 2008, **86**:101-110.

118.	**Shrestha RK *et al***. Cost-effectiveness of home-based chlorination and safe water storage in reducing diarrhea among HIV-affected households in rural Uganda. *American Journal of Tropical Medicine and Hygiene* 2006, **74**:884-890.

119.	**Sicuri E *et al***. Cost-effectiveness of intermittent preventive treatment of malaria in pregnancy in southern Mozambique. *PLoS ONE* 2010, **5**:e13407.

120.	**Sinha A *et al***. Cost-effectiveness of pneumococcal conjugate vaccination in the prevention of child mortality: an international economic analysis. *Lancet* 2007, **369**:389-396.

121.	**Sinha A *et al***. Cost-effectiveness of pneumococcal conjugate vaccination in Latin America and the Caribbean: a regional analysis. *Revista Panamericana de Salud Pública* 2008, **24**:304-313.

122.	**Skull SA, Butler JR**. Meningococcal vaccination for adolescents? An economic evaluation in Victoria. *Journal of Paediatrics and Child Health* 2001, **37**:S28-33.

123.	**Skull SA *et al***. Should programmes for community-level meningococcal vaccination be considered in Australia? An economic evaluation. *International Journal of Epidemiology* 2001, **30**:571-578; discussion 578-579.

124.	**Suarez PG *et al***. Feasibility and cost-effectiveness of standardised second-line drug treatment for chronic tuberculosis patients: a national cohort study in Peru. *Lancet* 2002, **359**:1980-1989.

125.	**Suaya JA *et al***. Cost-effectiveness of annual targeted larviciding campaigns in Cambodia against the dengue vector Aedes aegypti. *Tropical Medicine & International Health* 2007, **12**:1026-1036.

126.	**Sweat M *et al***. Cost-effectiveness of voluntary HIV-1 counselling and testing in reducing sexual transmission of HIV-1 in Kenya and Tanzania. *Lancet* 2000, **356**:113-121.

127.	**Sweat MD *et al***. Cost-effectiveness of nevirapine to prevent mother-to-child HIV transmission in eight African countries. *AIDS* 2004, **18**:1661-1671.

128.	**Sweat M *et al***. Cost-effectiveness of environmental-structural communication interventions for HIV prevention in the female sex industry in the Dominican Republic. *Journal of Health Communication* 2006, **11 Suppl 2**:123-142.

129.	**Terris-Prestholt F *et al***. Is antenatal syphilis screening still cost effective in sub-Saharan Africa. *Sexually Transmitted Infections* 2003, **79**:375-381.

130.	**Thielman NM *et al***. Cost-effectiveness of free HIV voluntary counseling and testing through a community-based AIDS service organization in Northern Tanzania. *American Journal of Public Health* 2006, **96**:114-119.

131.	**Torgerson PR *et al***. Alveolar echinococcosis: from a deadly disease to a well-controlled infection. Relative survival and economic analysis in Switzerland over the last 35 years. *Journal of Hepatology* 2008, **49**:72-77.

132.	**Touch S *et al***. A cost-effectiveness analysis of Japanese encephalitis vaccine in Cambodia. *Vaccine* 2010, **28**:4593-4599.

133.	**Tozan Y *et al***. Prereferral rectal artesunate for treatment of severe childhood malaria: a cost-effectiveness analysis. *Lancet* 2010, **376**:1910-1915.

134.	**Tupasi TE *et al***. Feasibility and cost-effectiveness of treating multidrug-resistant tuberculosis: a cohort study in the Philippines. *PLoS Medicine* 2006, **3**:e352.

135.	**Uruena A *et al***. Cost-effectiveness analysis of the 10- and 13-valent pneumococcal conjugate vaccines in Argentina. *Vaccine* 2011, **29**:4963-4972.

136.	**Uthman OA *et al***. The cost-utility analysis of adult male circumcision for prevention of heterosexual acquisition of HIV in men in sub-Saharan Africa: a probabilistic decision model. *Value Health* 2011, **14**:70-79.

137.	**Utzinger J *et al***. The economic payoffs of integrated malaria control in the Zambian copperbelt between 1930 and 1950. *Tropical Medicine & International Health* 2002, **7**:657-677.

138.	**Utzinger J *et al***. Efficacy and cost-effectiveness of environmental management for malaria control. *Tropical Medicine & International Health* 2001, **6**:677-687.

139.	**van Hulst M *et al***. Cost-effectiveness of HIV screening of blood donations in Accra (Ghana). *Value Health* 2008, **11**:809-819.

140.	**Varley RC *et al***. A reassessment of the cost-effectiveness of water and sanitation interventions in programmes for controlling childhood diarrhoea. *Bulletin of the World Health Organization* 1998, **76**:617-631.

141.	**Vega JC *et al***. Short communication: The cost-effectiveness of cutaneous leishmaniasis patient management during an epidemic in Chaparral, Colombia in 2004. *Tropical Medicine & International Health* 2007, **12**:1540-1544.

142.	**Vespa G *et al***. Estimating the cost-effectiveness of pneumococcal conjugate vaccination in Brazil. *Revista Panamericana de Salud Pública* 2009, **26**:518-528.

143.	**Vickerman P *et al***. Modelling the cost-effectiveness of introducing rapid syphilis tests into an antenatal syphilis screening programme in Mwanza, Tanzania. *Sexually Transmitted Infections* 2006, **82 Suppl 5**:v38-43.

144.	**Vickerman P *et al***. Are targeted HIV prevention activities cost-effective in high prevalence settings? Results from a sexually transmitted infection treatment project for sex workers in Johannesburg, South Africa. *Sexually Transmitted Diseases* 2006, **33**:S122-132.

145.	**Wang S *et al***. The Cost-Effectiveness of HIV Voluntary Counseling and Testing in China. *Asia-Pacific Journal of Public Health* 2011, **23**:620-633.

146.	**Wilkinson D *et al***. National and provincial estimated costs and cost effectiveness of a programme to reduce mother-to-child HIV transmission in South Africa. *South African Medical Journal* 2000, **Afr. med. j.. 90**:794-798.

147.	**Wilopo SA *et al***. Economic evaluation of a routine rotavirus vaccination programme in Indonesia. *Vaccine* 2009, **27 Suppl 5**:F67-74.

148.	**Wu F, Khlangwiset P**. Health economic impacts and cost-effectiveness of aflatoxin-reduction strategies in Africa: case studies in biocontrol and post-harvest interventions. *Food Additives and Contaminants Part a-Chemistry Analysis Control Exposure & Risk Assessment* 2010, **27**:496-509.

149.	**Xu Q *et al***. Cost effectiveness of DOTS and non-DOTS strategies for smear-positive pulmonary tuberculosis in Beijing. *Biomedical and Environmental Sciences* 2000, **13**:307-313.

150.	**Xu Q *et al***. Tuberculosis control priorities defined by using cost-effectiveness and burden of disease. *Biomedical and Environmental Sciences* 2002, **15**:172-176.

151.	**Yukich JO *et al***. Costs and cost-effectiveness of vector control in Eritrea using insecticide-treated bed nets. *Malaria Journal* 2009, **8**:51.

152.	**Zhang L *et al***. Needle and syringe programs in Yunnan, China yield health and financial return. *BMC Public Health* 2011, **11**.

153.	**Zinsstag J *et al***. Transmission dynamics and economics of rabies control in dogs and humans in an African city. *Proceedings of the National Academy of Sciences of the USA* 2009, **106**:14996-15001.
